# Excavation method optimization and mechanical responses investigating of a shallow buried super large section tunnels: a case study in Zhejiang

**DOI:** 10.1038/s41598-024-56982-7

**Published:** 2024-03-15

**Authors:** Yunteng Chen, Xiaoliang Geng, Jianjun Li, Mingfeng Zhang, Chengfeng Zhu, Mingcheng Cai, Wenlin Zhao, Xin Zhou, Tianzuo Wang

**Affiliations:** 1 Shaoxing Communications Investment Group Co., Ltd., Shaoxing, 312000 China; 2 China Construction Eighth Engineering Division Corp., Ltd., Shanghai, 200112 China; 3 Shaoxing Future Community Development and Construction Co., Ltd., Shaoxing, 312000 China; 4 Key Laboratory of Rock Mechanics and Geohazards of Zhejiang Province, School of Civil Engineering, Shaoxing University, Shaoxing, 312000 China

**Keywords:** Super large section tunnels, Mechanical responses, Construction optimization, Field monitoring, Numerical simulation, Engineering, Solid Earth sciences, Geology, Petrology

## Abstract

The construction of super large section (SLS) shallow buried tunnels involves challenges related to their large span, high flat rate, and complex construction process. Selecting an appropriate excavation method is crucial for ensuring stability, controlling costs, and managing the construction timeline. This study focuses on the selection of excavation methods and the mechanical responses of SLS tunnels in different types of surrounding rock. The research is based on the Yangjiashan tunnel project in Zhejiang Province, China, which is a four-line highway tunnel with a span of 21.3 m. Three sequential excavation methods were proposed and simulated using the three-dimensional finite difference method: the “upper first and lower later” side drift (SD) method, the central diaphragm method, and the top heading and bench (HB) method. The mechanical response characteristics of tunnel construction under these methods were investigated, including rock deformation, rock pressure, and the internal forces acting on the primary support. By comparing the performance of the three construction methods in rock masses of Grades III to V, the study aimed to determine the optimal construction method for SLS tunnels considering factors such as safety, cost, and schedule. Field tests were conducted to evaluate the effectiveness of the optimized construction scheme. The results of the field monitoring indicated that the “upper first and lower later” SD method in Grade V rock mass and the HB method in Grade III to IV rock mass are feasible and cost-effective under certain conditions. The research findings provide valuable insights for the design and construction of SLS tunnels in complex conditions, serving as a reference for engineers and project managers.

## Introduction

In recent years, China's economy has experienced continuous improvement, leading to an urgent demand for the development of highway transportation^1^. As a result, a tremendous amount of the single-hole four-lane super-large-section (SLS) tunnels have been extensively constructed to meet the traffic demand, including the Letuan Tunnel^2,3^, Jianzicha Tunnel^4^, Laohushan Tunnel^5,6^ and Xiabeishan tunnel^7,8^. Compared with the small and medium section tunnels, the SLS tunnels have larger space and higher traffic efficiency^3^. However, the increasing cross-section size and excavation area have given rise to more construction difficulties and larger disturbance to rock mass, resulting in extreme excavation risks during tunnel construction^4^. Improper tunnel design and excavation methods may lead to unforeseen incidents such as tunnel collapse, water seepage, and equipment failure, which could suddenly cause extensive ground subsidence, posing a serious threat to both traffic and personal safety^1^. To improve the stability of the tunnel face and reduce the construction risks, the sequential excavation methods (SEM) are widely employed in the SLS tunnels, such as the top heading and bench (HB) method, the central diaphragm (CD) method, the side drift (SD) method and the three-bench seven-step excavation method^9–12^. The excavation methods have a significant impact on the stability, the cost and time of tunnel construction^9,11^. Thus, the selection of the excavation method is considered as a key challenge for the effective and safe construction of the SLS tunnel^11^.

In the last decades, a large number of studies have been made to the selection of SEM using theoretical analysis, numerical simulation and field monitoring^6^. For example, Hoek^13^ and Yu and Chern^14^ proposed two graphs method based on strength factor—strain and strength factor—tunnel width for determining the SEM through numerical analyses and case studies. Sharifzadeh et al.^11^ conducted a series of 3D finite element analyses for selection of excavation method, optimal excavation sequences and trailing distance between different excavations faces based on its potential to limit surface settlements. Daraei and Zare^9^ proposed a new multi-graph approach for selecting the SEM of the tunnels, based on some parameters such as failure strain, modified secant modulus, strength factor and the tunnel span to determine the sequential excavation method. Galli et al.^15^ developed a three-dimensional numerical model to simulate the process of tunnel excavation and support construction. Their findings indicated that the attributes of the rock mass properties and the excavation steps were the primary factors influencing tunnel deformation and surface settlement.

Although a reasonable excavation method is essential for successful construction, the mechanical responses of surrounding rock and supporting structures under various excavation method are also crucial for tunnel construction^1^. Chen et al.^2^ and Luo et al.^3^ conducted a study on the deformation behaviors and mechanical properties of SLS and shallow tunnel constructed through the upper-bench CD method, employing numerical modeling and back-calculation techniques. Their findings highlighted a significant influence of construction method and support structure on tunnel deformation and rock mass pressure. Luo et al.^4^ examined the spatio-temporal behavior of rock mass deformation and the stress state of the supporting structure in large-span loess tunnels. Their research uncovered a three-phase deformation pattern, characterized as 'rapid deformation stage—continuous deformation stage—slow deformation stage', during the construction process utilizing the annular excavation with reserved core soil method. He et al.^7^ and Ma et al.^8^ investigated the mechanical responses and construction optimization for shallow SLS tunnels in weathered tuff stratum constructed through the SD method based on field monitoring and FLAC 3D modeling. The results showed that the construction disturbance is mainly concentrated in the excavation of the upper benches and the excavation of the upper bench of the middle heading is the most critical construction step.

The above studies have significantly enriched the selection of the SLS excavation method, improving the level of tunnel design, and ensuring the safety of tunnel construction. However, due to the complicated excavation steps in the SEM, the interactions between surrounding rock and supporting structures of SLS tunnels are extremely elusive. The ground surface settlement and supporting structure deformation induced by tunnelling varies according to different excavation methods used for different tunnel cross sections^9^. Therefore, decision-making about appropriate excavation methods for SLS tunnels should take into account of the adaptability and economy of different excavation methods to the different surrounding rock conditions^11^. In this paper, the construction schemes for a shallow four-line highway tunnel with a span of 21.3 m are investigated by employing numerical analysis and in situ tests. First, the ground displacement and support stress characteristics of the shallow SLS tunnel in Grades III–V rock mass with different construction approaches were comprehensively compared and analyzed. Subsequently, the optimized construction schemes for the SLS tunnel in different rock mass condition were determined based on the safety, cost and schedule considerations. Finally, the optimized effect of the excavation scheme was checked by field monitoring and the evolution law of tunnel deformation was discussed. This paper provides an in-depth illustration of the effect of the construction schedule on tunnelling performance which helps us to select excavation method in a cost-effective way.

## Project overview

**Figure 1:**
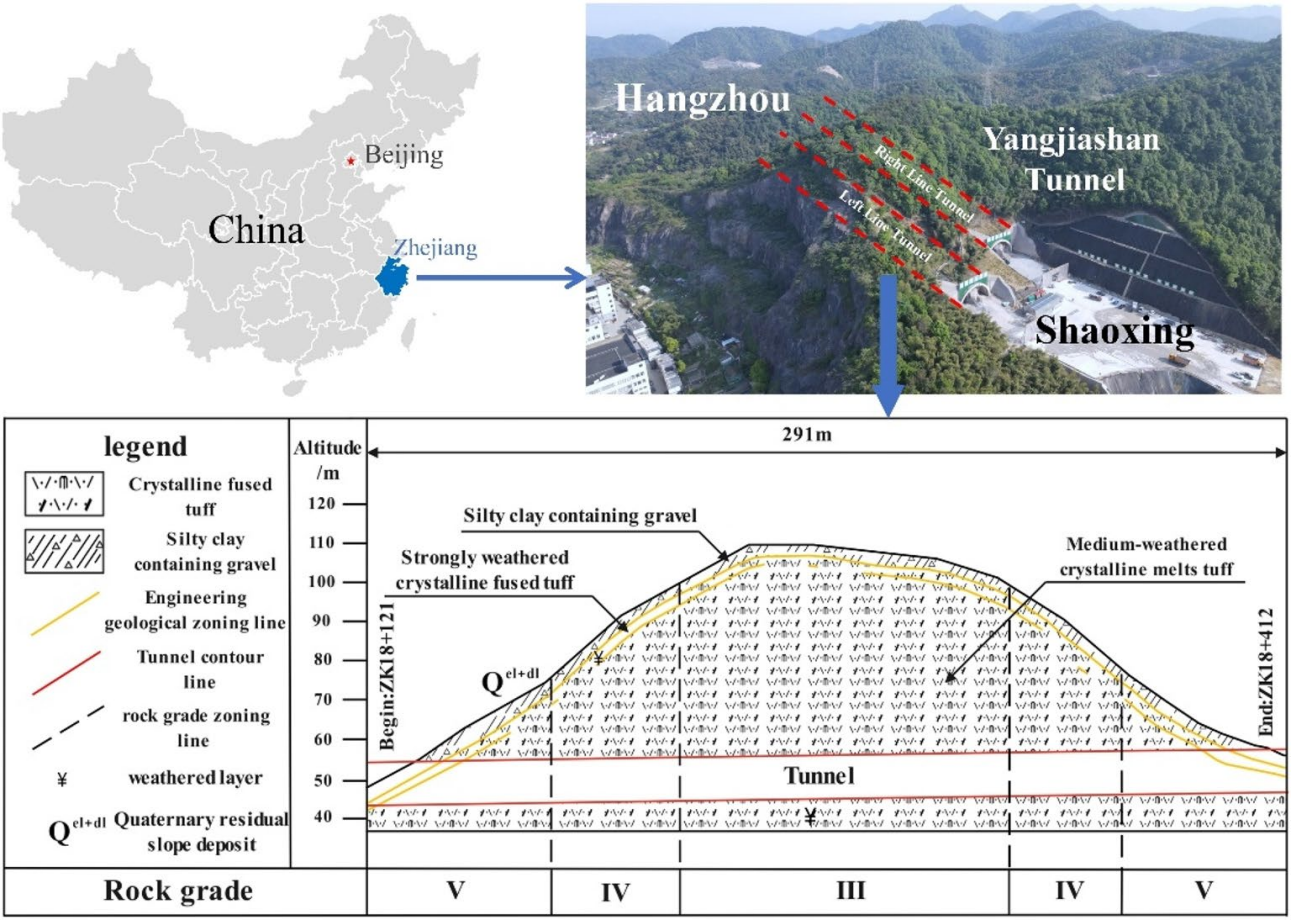
Location of the tunnel in China, and the geological profile of tunnel site.

As shown in Fig. 1, the Yangjiashan Tunnel, a vital component of the Hangzhou-Jinhua-Quzhou high-speed connection line, is located in Shaoxing City, Zhejiang Province, China. This tunnel, designed as a two-hole, eight-lane shallow SLS structure (the area of the tunnel cross-section > 100 m^2^), has a maximum excavated width of 21.3 m and a maximum excavated area of 249.25 m^2^. The left line of the tunnel is from ZK18 + 121 to ZK18 + 412 with a length of 291 m, and the right line is from YK18 + 154 to YK18 + 460 with a length of 306 m. The tunnel support is a composite liner consisting of a primary support and a secondary lining. The tunnel primary support parameters are listed in Table 1.

**Table 1:** Tunnel primary support parameters.

Rock grade	C30 shotcrete thickness (cm)	Systematic bolts (from arch to side wall)		Steel arch	
		Type, length (L) (m)	Spacing (cm)	Type	Spacing (cm)
V	32	φ25 hollow grouting bolt, L = 5.5 m	60 × 120	I25b	60
IV	28	φ25 hollow grouting bolt, L = 4 m	100 × 120	I20b	100
III	25	φ22 cement mortar bolt, L = 3.5 m	120 × 120	I18	120

The tunnel site is located in a hilly terrain characterized by low hills. The strata of the tunnel primarily consist of the Chalky Shougang Formation (K1s) crystalline fused tuff. The entrances and exits of the left and right line tunnels are all situated on the slope of the hill, surrounded by 3–5 m thick, strongly weathered tuff. The tunnel is shallowly buried, with a maximum depth of 61 m, not exceeding three times the tunnel diameter. A significant portion of the tunnel is buried at a depth less than twice the tunnel diameter. Consequently, excavation may lead to increased risks of ground and structural instability. The overall geological conditions are challenging, with the development of joint cracks in the local surrounding rock segments. According to the standard for engineering classification of rock mass (GBT50218-2014), the rock mass along the tunnel entrance could be classified as Grades V, IV, and III, as illustrated in Fig. 1.

## Numerical analysis of the optimization of excavation methods

### Excavation methods

**Figure 2:**
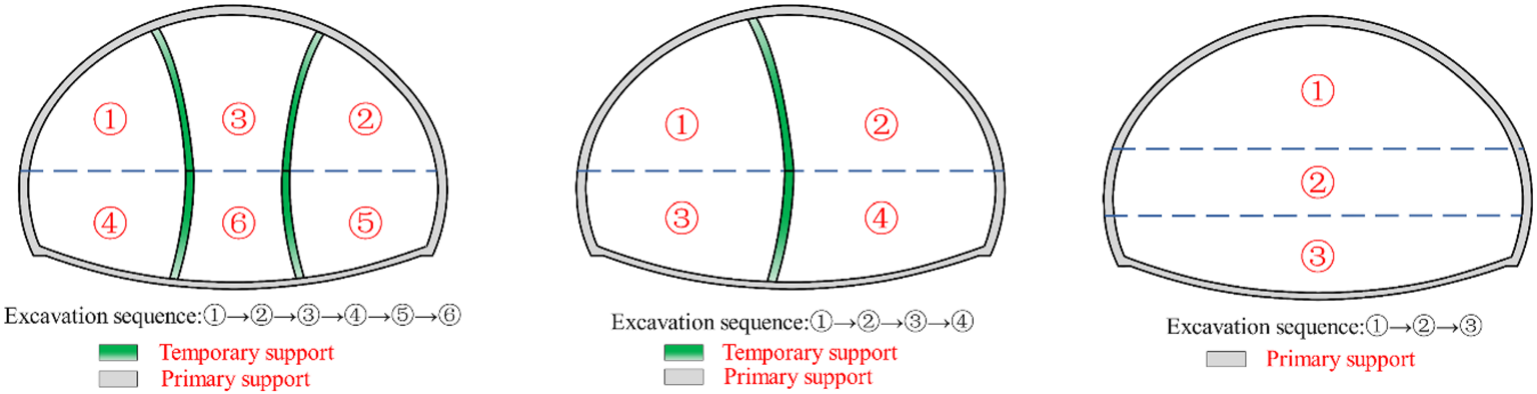
Tunnel excavation sequence for the SD, CD and HB excavation methods.

According to the SEM, the construction of super large section (SLS) tunnels involves complex excavation and support procedures that can significantly disturb the surrounding rock mass. Therefore, selecting the appropriate excavation methods and sequencing schemes for SLS tunnels should be based on a comprehensive evaluation of construction safety, cost, and time. Currently, the SD, CD, and HB methods are widely acknowledged as the most suitable excavation techniques for SLS tunnels^9,11^. Thus, to determine the most appropriate excavation method for different rock mass conditions, a comparative analysis of these excavation methods was conducted using the numerical finite difference method in this section. Figure 2 depicts the tunnel excavation sequences for the SD, CD, and HB methods used in the numerical simulation. It is worth noting that the excavation sequences employed in this study for the SD and CD methods differ from the traditional approaches of these methods. The modified excavation sequence, which involves excavating the upper portion first and the lower portion later, was adopted based on the lessons learned from on-site construction. Compared with the traditional SD and CD method, this modified sequence can greatly improve the excavation efficiency.

### Numerical model and calculation parameters

**Figure 3:**
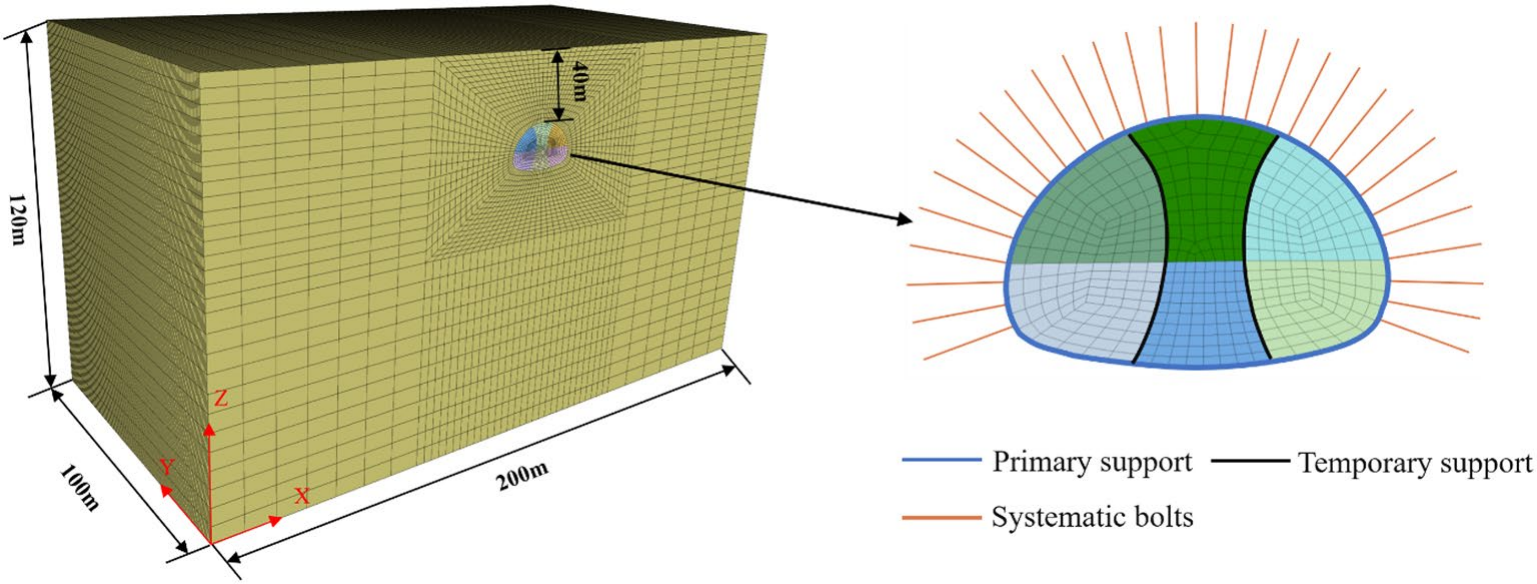
Numerical modelling by Flac3D.

The numerical model for the SLS tunnel is presented in Fig. 3, taking Grade V rocks as a representative example for illustration. Based on Fenner’s solution^16^, the tunnel construction has a limited impact on the surrounding rock beyond a distance of five times the tunnel radius. In order to minimize the influence of boundary effects on the simulation results, the left and right boundaries as well as the lower boundary of the model are established at a distance of 5 times the tunnel radius. The dimensions of the Yangjiashan tunnel model, including width (X), thickness (Y), and height (Z), are set to 200 m, 100 m, and 120 m, respectively. The bottom and four sides of the model are imposed with fixed and normal constraints, respectively, while the top of model is assigned as a free surface.

**Figure 4:**
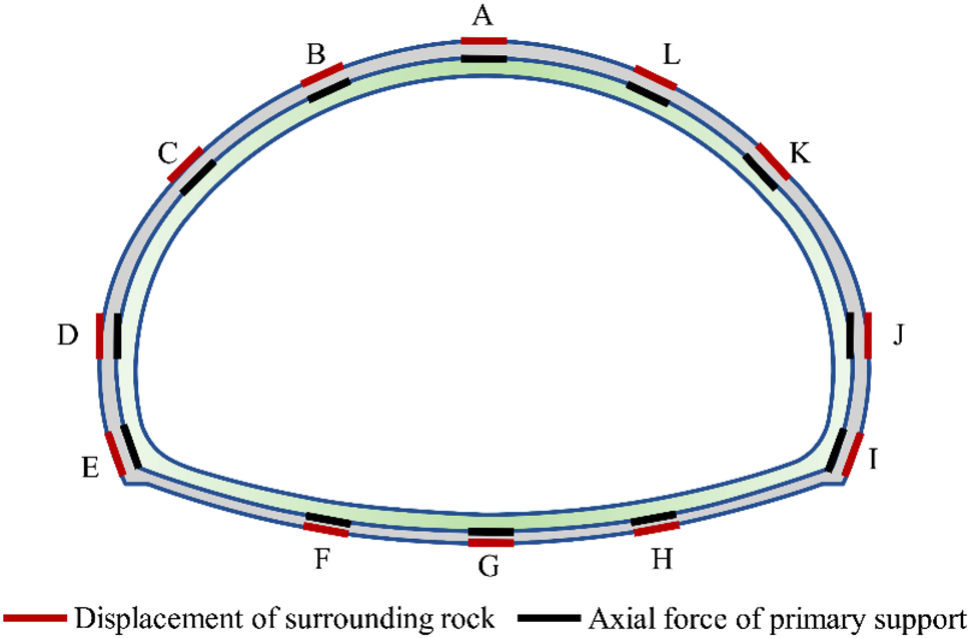
Measuring points arrangement.

The Mohr–Coulomb model is a constitutive model that is widely used in geotechnical engineering for simulating rock and soil behavior due to its simplicity and effectiveness in representing the shear strength of geological materials^17,18^. Therefore, the surrounding rock is simulated by solid elements and followed the Mohr–Coulomb constitutive model. The physical and mechanical parameters of the surrounding rock, based on the geological investigation data of the Yangjiashan Tunnel project, are presented in Table 2. In the numerical analysis, the primary and temporary support are taken into account and simulated using shell elements. However, the secondary lining is excluded from the analysis because it is usually positioned at a significant distance from the excavation face and serves as a safety reserve^11^. The calculation parameters of the supporting structures are determined by the equivalent reduction of steel arch and shotcrete. The systematic bolts are represented by cable elements in the simulation, and the calculation parameters for these supporting structures can be found in Tables 3 and 4. The tunnel excavation is performed using the model null command. The excavation of the tunnel is achieved with the model null command, and the excavation footage for the rocks of Grades V, IV, and III are 1.2 m, 2 m, and 3 m, respectively. To analyze the deformation and stress characteristics of the surrounding rock and supporting structures in the tunnel, twelve axial force of primary support monitoring points and twelve displacement of surrounding rock monitoring points are chosen in the monitoring section. These points are strategically positioned at key locations such as the vault, waist and sidewall, as illustrated in Fig. 4.

**Table 2:** Physical and mechanical parameters of surrounding rock.

Rock grade	Density (kg m^−3^)	Elastic modulus (GPa)	Possion’s ratio	Cohesion (MPa)	Friction angle (°)
V	2000	1	0.37	0.1	25
IV	2300	3	0.33	0.2	33
III	2600	6	0.28	0.7	40

**Table 3:** Calculation parameters of primary support structures.

Rock grade	Density (kg m^−3^)	Elastic modulus (Gpa)	Possion’s ratio	Thickness (mm)
V	2500	28	0.2	32
IV	2420	25.5	0.2	28
III	2400	25	0.2	25

**Table 4:** Calculation parameters of Systematic bolts.

Elastic modulus (GPa)	Possion’s ratio	Grout stiffness (kN m^−2^)	Grout cohesion (MPa)	Grout friction (°)
210	0.2	2.7 × 10^6^	2.6	45

### Results and discussion of the numerical analysis

#### Deformation analysis of surrounding rock

##### Grade V rock mass

**Figure 5:**
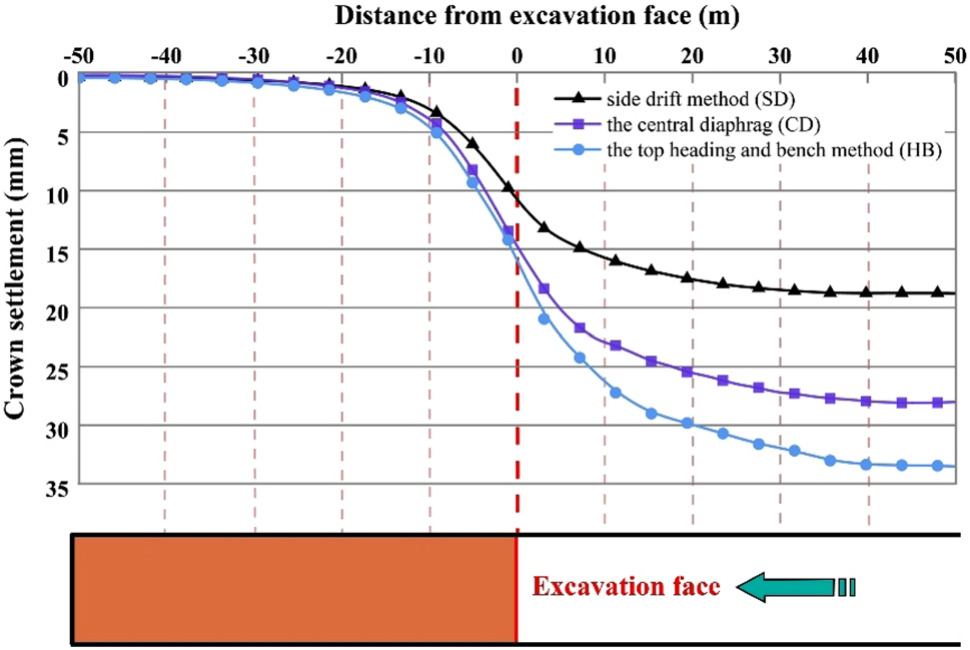
Longitudinal vault settlement profiles for the SD, CD and HB excavation methods in Grade V rocks.

The longitudinal vault settlement profiles for different excavation methods in Grade V rocks are illustrated in Fig. 5. The X-axis represents the distance between the excavation face and the monitoring section, while *u*_a_ and *u*_s_ indicate the pre-excavation advanced deformation of the unexcavated portion and the final deformation of the SLS tunnel, respectively. As depicted in Fig. 5, the longitudinal changes in vault settlement exhibit a consistent pattern across various excavation methods. Initially, there is a gradual increase in advanced deformation, followed by a rapid increase, and finally reaching a stable deformation stage. This observed trend aligns with the vertical displacement development pattern documented in previous studies^1^.

A comparison of the longitudinal vault settlement profiles of different excavation methods indicates that the selected method has a significant influence on the spatial deformation effect on the surrounding rock. Generally, a reduced number of excavation steps in the construction of the SLS tunnel leads to a greater extent of disturbance to the surrounding rock. Specifically, the HB method exhibits the largest disturbance distance, with vault settlement occurring up to 46 m ahead of the tunnel face and reaching stability at 45 m behind it. In contrast, the SD and CD methods result in disturbance distances of 42 m and 44 m before tunnel excavation, and 35 m and 40 m behind the tunnel face, respectively. These findings are consistent with the statements made by Carranza-Torres and Fairhurst^19^ and Sharifzadeh et al.^11^, who reported that excavation influence can be disregarded when the distance behind the tunnel face is more than 4D and the distance in front of the tunnel face is more than 3D. Here, "D" represents the tunnel's equivalent radius, which is 9 m in this particular study. Additionally, it is worth noting that the average ratio of the vault settlement above the tunnel face to the final settlement for different excavation methods, represented by *u*_a_/*u*_s_, is approximately 52%. This result aligns with the simulation results provided by Sharifzadeh et al.^11^, indicating the importance of implementing appropriate advanced support measures to effectively control tunnel deformation.

**Figure 6:**
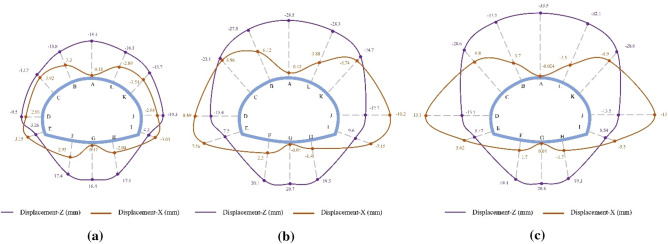
Final displacement distributions for the SD, CD and HB excavation methods in Grade V rocks.

The final displacement distributions of the SLS tunnel for the SD, CD, and HB excavation methods in Grade V rocks are presented in Fig. 6. The displacement distribution characteristics are generally consistent among these three construction methods. Vertical displacement is symmetrically distributed along the axis of the SLS tunnel and reaches its maximum at the vault and inverted arch (i.e., points A and G). Similarly, the horizontal displacement also exhibits a symmetrical distribution, with the maximum horizontal convergence predominantly observed in the side wall (i.e., points D and J). In the CD and HB methods, the maximum vault settlements are approximately 28.8 mm and 33.5 mm, respectively, accompanied by corresponding horizontal convergences of 10.2 mm and 13.1 mm. These values are higher than the maximum vault settlement (18.4 mm) and horizontal convergence (3.92 mm) of the SD method. This suggests that as the number of excavation stages increases in SLS tunnel construction, rock deformation in Grade V rocks decreases. Therefore, the SD method is preferable in Grade V rocks due to its effectiveness in limiting tunnel rock deformations. Furthermore, the horizontal convergence of the tunnel is significantly smaller compared to the vault settlement. This phenomenon can be attributed to the low flat rate of the SLS tunnel, which causes the surrounding rock at the upper part of the tunnel to exert pressure on the arch support, while the sidewall support is pressed against the surrounding rock by the force transmitted from the arch support^1^.

##### Grade III and IV rock mass

**Figure 7:**
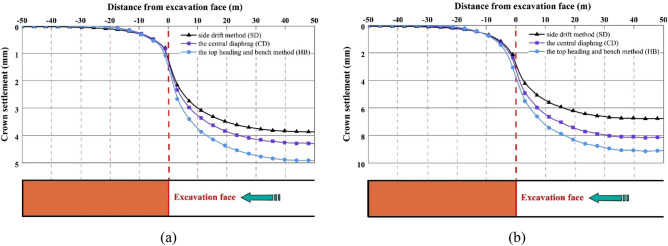
Longitudinal vault settlement profiles for the SD, CD and HB excavation methods in Grade III and IV rocks.

The longitudinal vault settlement profiles for different excavation methods in Grade III and IV rocks are depicted in Fig. 7. The development characteristics of these profiles in Grade III and IV rocks are similar to those observed in Grade V rocks, exhibiting an S-shaped evolutionary pattern. The extent of disturbance to the surrounding rock caused by tunnel excavation in Grade III and IV rocks is relatively smaller compared to Grade V rocks. The advanced disturbance distances in Grade III and IV rocks are approximately 25 m and 30 m, respectively, with corresponding advanced disturbance distances behind the tunnel face of 35 m and 37 m. Additionally, the average ratio of the vault settlement above the tunnel face to the final settlement for different excavation methods in Grade III and IV rocks is 30% and 40%, respectively. These values are significantly lower than the average ratio of 52% observed in Grade V rocks. These findings indicate that the extent of excavation disturbance around the tunnel face primarily depends on the ground conditions, which has been also reported by Sharifzadeh et al.^11^. The higher the grade of the surrounding rock, the smaller the disturbance distance and advanced convergence of construction methods on rock deformation. Therefore, it is recommended to implement advance reinforcement measures such as advance small pipe grouting reinforcement, deep hole grouting reinforcement, and pipe shed support before excavation to limit advanced convergence and maintain tunnel stability when constructing SLS tunnels in soft or weak geological strata^20,21^.

**Figure 8:**
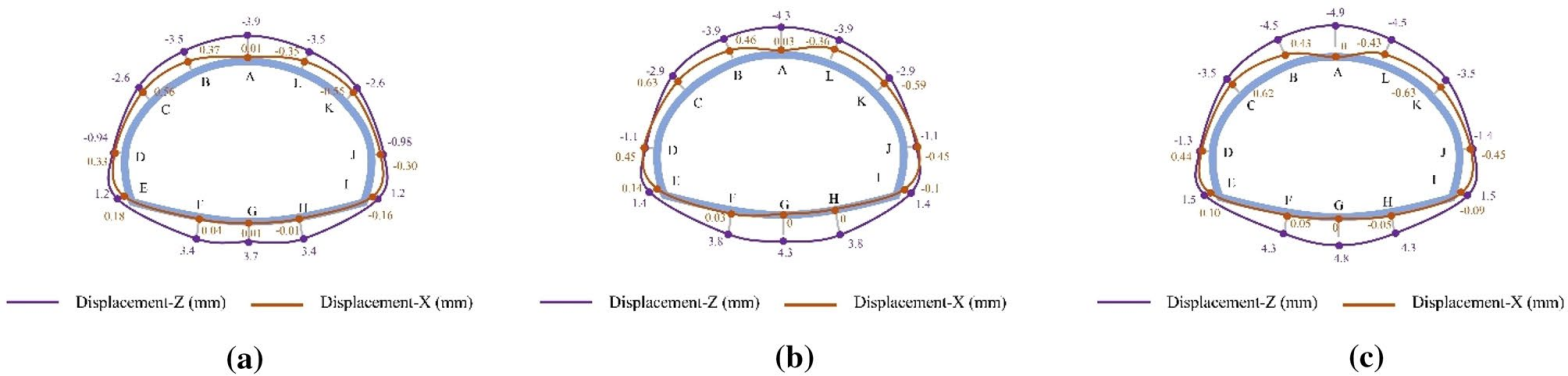
Final displacement distributions for the SD, CD and HB excavation methods in Grade III rocks.

**Figure 9:**
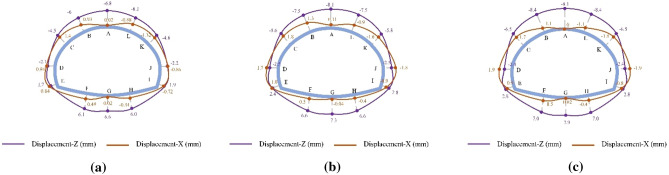
Final displacement distributions for the SD, CD and HB excavation methods in Grade IV rocks.

The final displacement distributions of the SLS tunnel for the SD, CD, and HB excavation methods in Grade III and IV rocks are illustrated in Figs. 8 and 9. Similar to the displacement distribution characteristics observed in Grade V rocks, the maximum rock subsidence for the SD, CD, and HB excavation methods in Grade III and IV rocks is located at the tunnel vault, while the largest horizontal convergence occurs in the tunnel wall waist. The shallow SLS tunnel employing the SD method exhibits the smallest rock deformation, with vault settlements of 3.9 mm in Grade III rocks and 6.8 mm in Grade IV rocks, as well as horizontal convergences of 0.56 mm in Grade III rocks and 1.4 mm in Grade IV rocks. This is followed by the CD method, which has vault settlements of 4.3 mm in Grade III rocks and 8.1 mm in Grade IV rocks, along with horizontal convergences of 0.63 mm in Grade III rocks and 1.8 mm in Grade IV rocks. The HB method results in vault settlements of 4.9 mm in Grade III rocks and 9.1 mm in Grade IV rocks, as well as horizontal convergences of 0.63 mm in Grade III rocks and 1.9 mm in Grade IV rocks. These findings indicate that the SD method, with fewer excavation steps, leads to less rock disturbance. However, with improved rock conditions, the differences in rock deformation between different excavation methods gradually decrease. Consequently, the excavation method has minimal influence on the rock displacement of shallow SLS tunnels in Grades III and IV rocks, which differs significantly from Grade V rocks. Considering construction efficiency and the stability of the surrounding rock, the HB method is recommended for constructing SLS tunnels in favorable geological stratum conditions.

#### Stress analysis of surrounding rock

##### Grade V rock mass

**Figure 10:**
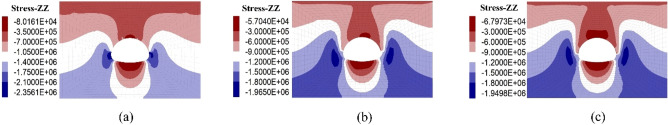
Final vertical stress distribution in the surrounding rock for the SD, CD and HB excavation methods in Grade V rocks.

Figure 10 illustrates the final distribution of vertical stress in the surrounding rock when applying three excavation methods (SD, CD, and HB) to Grade V rocks. Negative values indicate compressive stress. As shown in Fig. 10, all three construction methods result in a symmetrical stress field in the surrounding rock, with stress unloading at the vault and inverted arch, and stress concentration on both sides of the tunnel wall. When employing the SD method in SLS tunnels, the maximum vertical stress in the surrounding rock, approximately 2.35 MPa, concentrates at the sidewalls on both sides of the tunnel. In contrast, the CD and HB methods yield significantly lower vertical stresses in the same area, which are approximately 1.04 MPa and 0.56 MPa, respectively. Moreover, the SD and HB methods lead to a notable expansion of the stress concentration area, which shifts to a deeper region of the surrounding rock sidewalls. This shift occurs because the stress release extent of the surrounding rock is considerably higher for the SD and HB methods compared to the SD method. As a result, the surrounding rock undergoes plastic yield, and the stress is transferred to deeper regions. Consequently, the SD method proves more advantageous in redistributing stress within the surrounding rock during the construction of SLS tunnels in weak geological strata.

##### Grade III and IV rock mass

**Figure 11:**
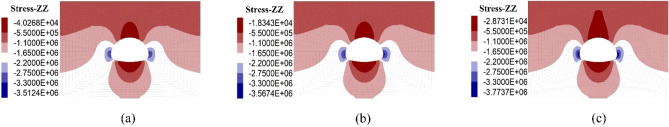
Final vertical stress distribution in the surrounding rock for the SD, CD and HB excavation methods in Grade III rocks.

**Figure 12:**
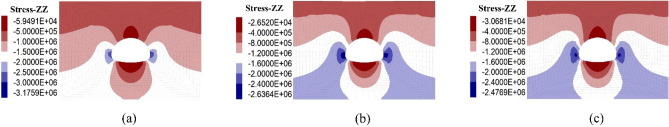
Final vertical stress distribution in the surrounding rock for the SD, CD and HB excavation methods in Grade IV rocks.

Figures 11 and 12 depict the final distribution of vertical stress in the surrounding rock when applying three excavation methods (SD, CD, and HB) to Grade III and IV rocks. Similarly, negative values indicate compressive stress. As shown in Figs. 11 and 12, the stress distribution characteristics in the surrounding rock of SLS tunnels constructed in Grade III and IV rocks are similar to those of Grade V rocks. Stress unloading occurs at the vault and inverted arch, while stress concentration is predominantly observed in the sidewalls. When constructing SLS tunnels in Grade IV rocks, the maximum vertical stresses in the surrounding rock are 3.18 MPa, 2.63 MPa, and 2.48 MPa for the SD, CD, and HB methods, respectively. The extent of the stress concentration area gradually decreases with the increasing excavation steps of the construction method, consistent with the behavior observed in Grade V rocks. However, when constructing SLS tunnels in Grade III rocks, the differences in the stress concentration area between these three construction methods are significantly smaller. The maximum vertical stresses in the surrounding rock for the SD, CD, and HB methods are 3.51 MPa, 3.57 MPa, and 3.77 MPa, respectively, exhibiting an opposite trend compared to Grade IV and V rocks. This result suggests that the stress in the surrounding rock depends on the interaction between the rock and support and is closely related to the surrounding rock conditions^22^. When the surrounding rock is of poor quality, fewer excavation steps lead to more stress release, resulting in stress reduction and transfer to the deeper regions of the surrounding rock. Conversely, when the surrounding rock properties are good, fewer excavation steps and more stress release are more conducive to fully utilizing the self-bearing capacity of the surrounding rock.

#### Axial force analysis of primary support

##### Grade V rock mass

**Figure 13:**
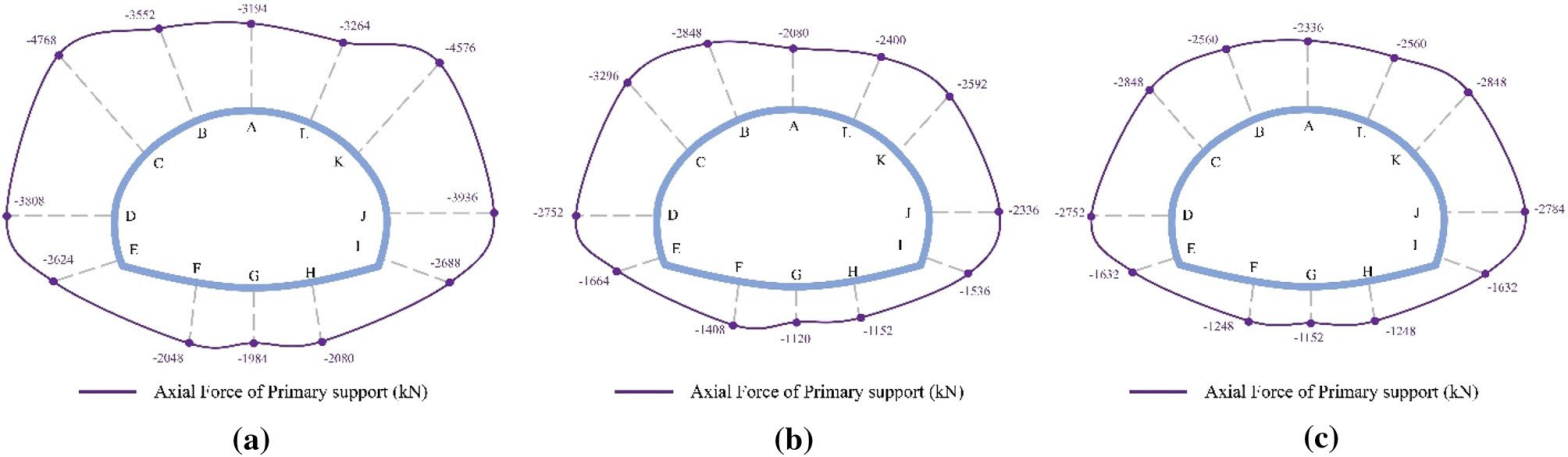
Final axial force distribution of the primary support for the SD, CD and HB excavation methods in Grade V rocks.

The final distribution of axial forces on the primary support under the SD, CD, and HB excavation methods in Grade V rocks is displayed in Fig. 13, where negative values indicate compression force. As shown in Fig. 13, the axial force of the primary support under three excavation methods exhibits similar distribution characteristics, with the primary support predominantly subjected to pressure. The axial force distribution of the primary support is symmetrical due to the symmetrical tunnel excavation method. It is mainly concentrated in the arch waist (points C and K) and side walls (points D and J), while the axial forces in the tunnel arch and inverted arch (points A and G) are relatively small, demonstrating a distribution pattern of "large in arch waist and side walls, small in arch and inverted arch." This trend aligns well with the shotcrete stress distribution pattern reported by He et al. based on the field monitoring results. The maximum axial force of the SD method is 4768 kN, significantly larger than that of the CD method (3296 kN) and HB method (2848 kN). This finding suggests that the stress release extent of the surrounding rock in the CD and HB methods is considerably higher than that in the SD method, which is the primary reason for the lower axial force on the primary support under the CD and HB methods compared to the SD method. Therefore, for tunnels constructed in weak geological strata that require timely support, the SD method is optimal to prevent excessive stress release and instability in the surrounding rock.

##### Grade III and IV rock mass

**Figure 14:**
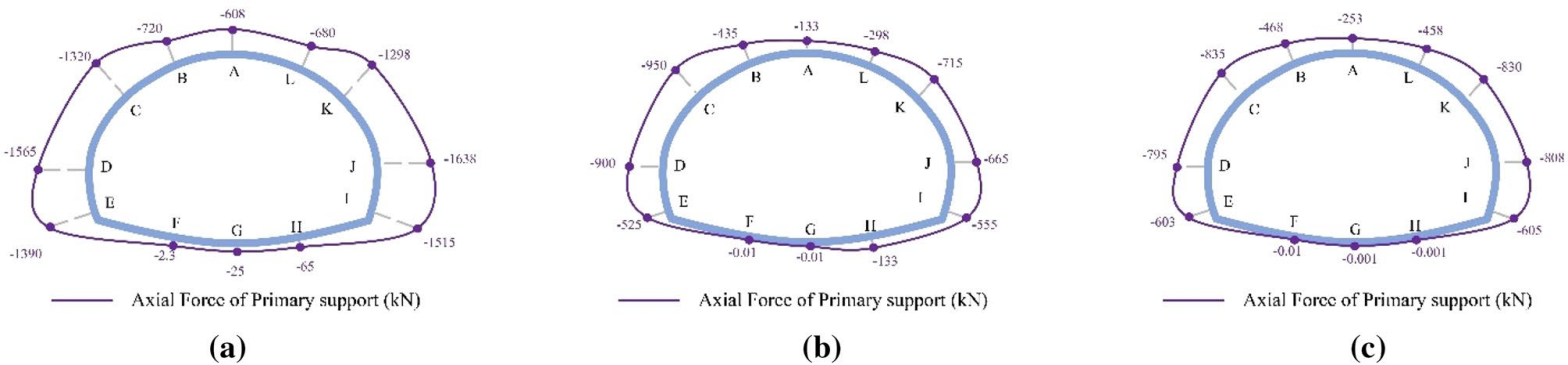
Final axial force distribution of the primary support for the SD, CD and HB excavation methods in Grade III rocks.

**Figure 15:**
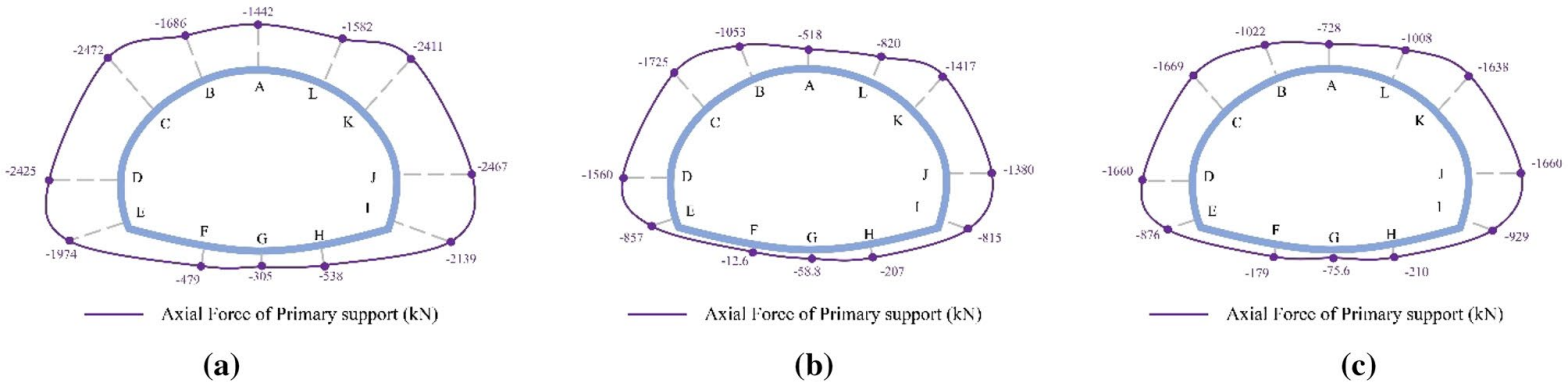
Final axial force distribution of the primary support for the SD, CD and HB excavation methods in Grade IV rocks.

The final distribution of axial forces on the primary support under the SD, CD, and HB excavation methods in Grade III and IV rocks is presented in Figs. 14 and 15. Similar to the axial force distribution in Grade V rock mass, the axial forces in Grade III and IV rocks for all three methods are mainly concentrated in the arch waist and side walls. The highest axial forces on the primary support are observed in Grade III (1638 kN) and IV rocks (2472 kN) when the SD method is used, followed by the CD method and the HB method. However, these values are significantly lower than the axial forces observed in Grade V rocks (4768 kN). These findings highlight the substantial influence of surrounding rock conditions on the axial forces experienced by the primary support in SLS tunnels. The worse the surrounding rock condition, the greater the axial force on the primary support. Therefore, when employing the SD method for constructing shallow SLS tunnels in weak ground, measures such as increasing the shotcrete strength grade and improving the distribution density of steel arches should be implemented to enhance the compressive bearing capacity of the primary support and prevent compressive failure. Furthermore, the difference in maximum axial forces on the primary support among the three methods in Grade III (1638–835 kN) and IV rocks (2472–1669 kN) is relatively smaller compared to Grade V rocks (4768–2848 kN). These results suggest that the choice of excavation method has minimal influence on the axial force experienced by the primary support in Grade III and IV rocks. Therefore, the HB method is the optimal method for constructing SLS tunnels in Grade III and IV rocks, as it can utilize the self-bearing capacity of the surrounding rocks and promote a favorable stress state for the primary support structure.

### Optimized excavation method for SLS tunnels

Based on a comprehensive analysis of the deformation and stress of surrounding rock, axial force of the primary support, construction conditions, and construction progress, an optimized excavation method can be determined for the SLS tunnel. In areas where Grade V rocks are present at both ends of the tunnel, posing a relatively high safety risk during construction, the evaluation of rock deformation and primary supporting axial force indicates that selecting the modified SD construction method ("upper first and lower later" side drift method), can effectively control surrounding rock deformation and meet safety requirements. Therefore, it is recommended to use the SD method for excavating sections with Grade V rocks. On the other hand, for sections with Grade III–IV rocks, the HB method can be considered due to its simplified construction process, higher excavation efficiency, and lower construction costs. It is worth noting that the HB method has already been successfully implemented in the Badaling Great Wall Station^17,18^ and the Wufengshan No.2 Tunnel^23^.

## Field monitoring

**Figure 16:**
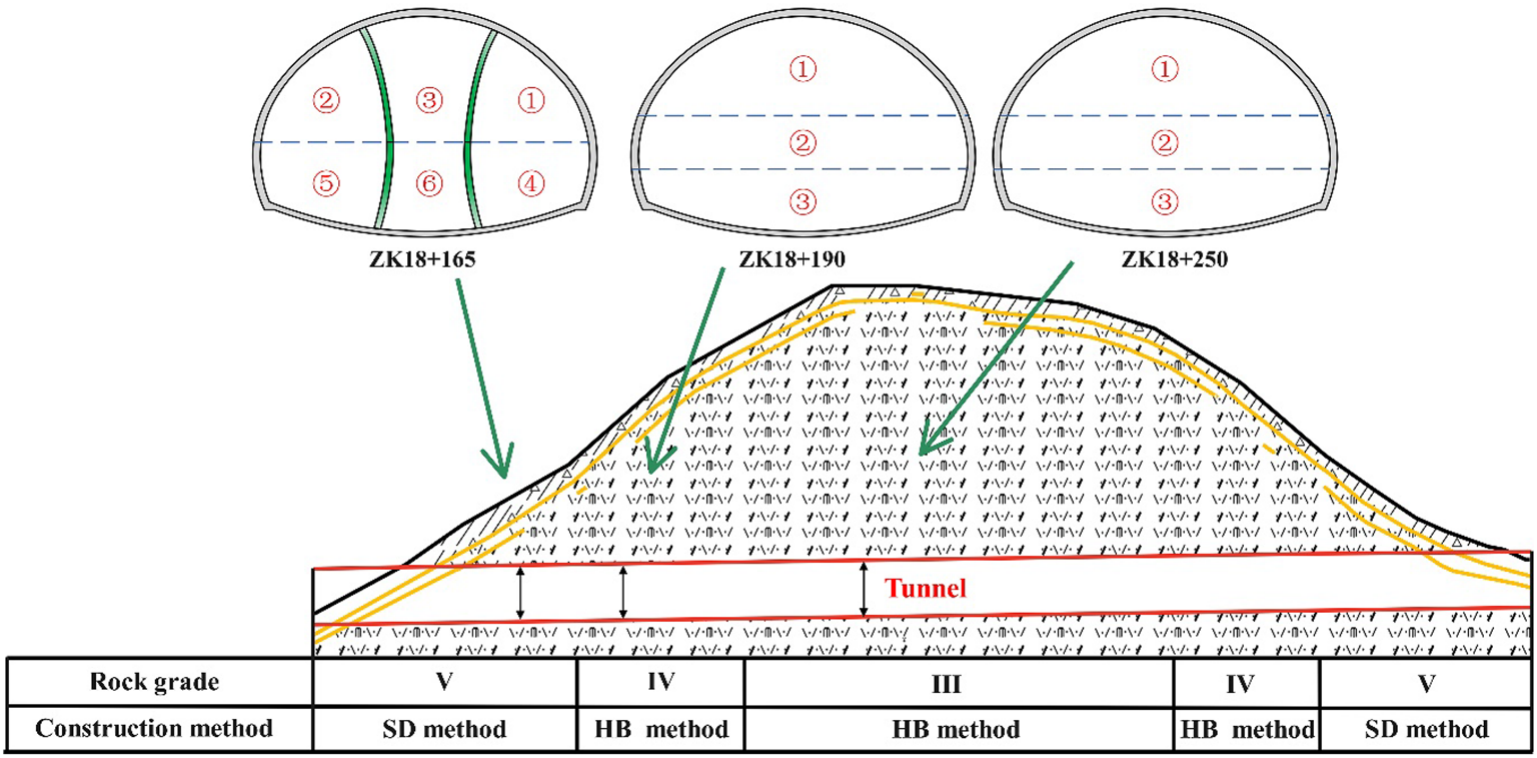
Excavation methods for the SLS tunnel and field monitoring sections.

Based on numerical analysis, optimized methods for the SLS tunnel were determined and implemented in field construction. Figure 16 illustrates the implementation of the SD and HB methods in the Grade V rock section and Grade III and IV rock sections, respectively. Field monitoring of deformation was conducted at sections ZK18 + 168, ZK18 + 190, and ZK18 + 250 to verify the feasibility of the proposed scheme.

**Figure 17:**
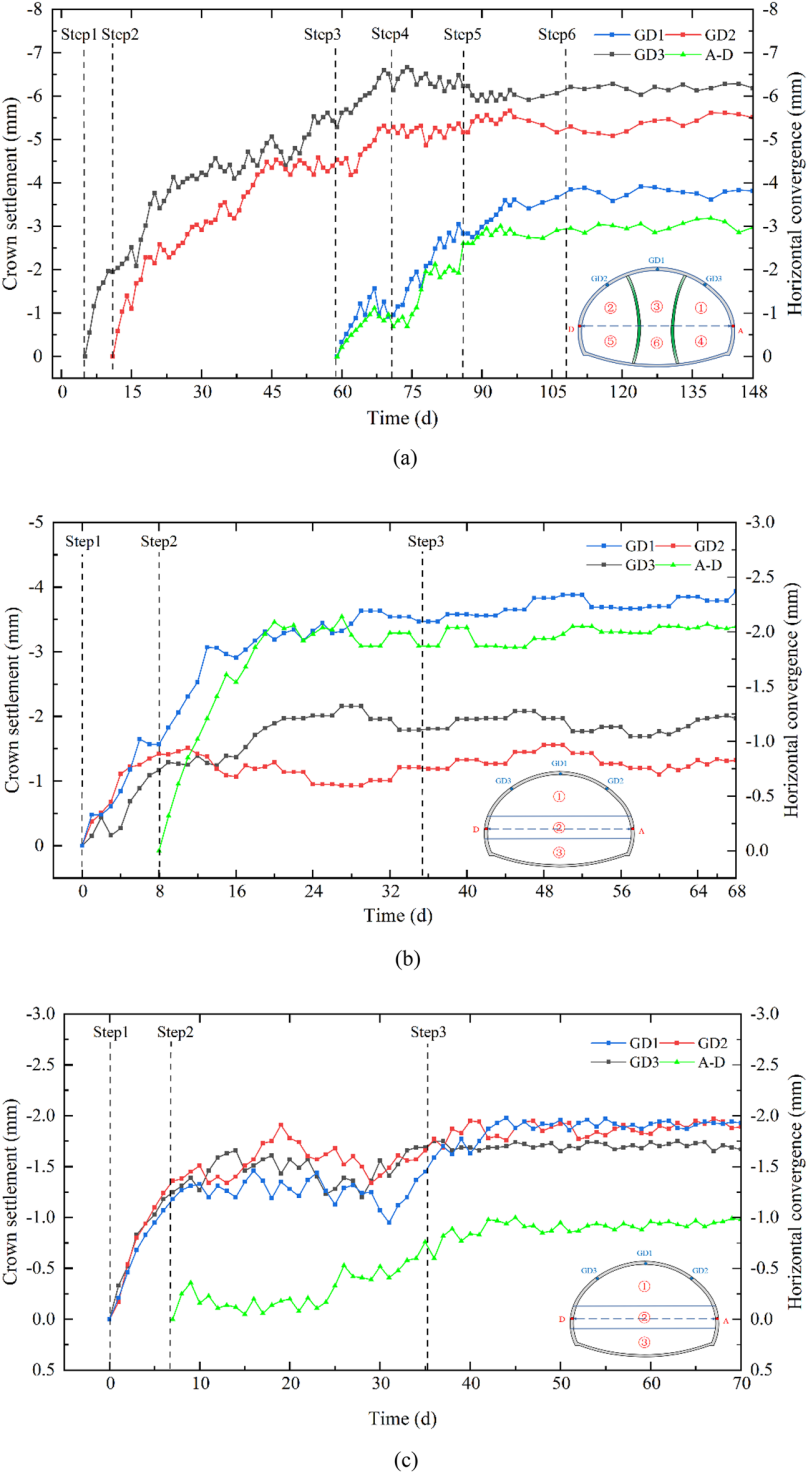
Development curves of the tunnel vault settlement and horizontal convergence.

Figure 17a illustrates the development curves of vault settlement and horizontal convergence in section ZK18 + 168, where the inward deformation of the tunnel is negative. As depicted in Fig. 17a, the vertical displacement of the three monitoring points (GD1, GD2, and GD3) exhibits similar characteristics. The vertical displacements experience a significant increase following the excavation of the upper benches in each pilot tunnel (i.e., S1, S2, and S3) and subsequently stabilize within approximately 30 days (36 m behind the tunnel face), aligning well with the numerical simulation. This phenomenon suggests that the excavation of the upper bench in the right, left, and middle pilot tunnels represents a crucial construction step, as the stress adjustment of the surrounding rock primarily occurs during the initial excavation stage. The left pilot tunnel exhibits the greatest final vertical displacement (6.66 mm), larger than that of the right pilot tunnel (5.66 mm) and the middle tunnel (3.9 mm). This can be attributed to the disturbance effect of the pilot tunnel excavated at a later stage on the pilot tunnel excavated earlier. The horizontal convergence exhibits a comparable development trend to the vault settlement after the excavation of the tunnel's lower bench, with the final values being approximately 3 mm smaller.

The development curves of vault settlement and horizontal convergence in section ZK18 + 168 and are shown in The development curves of vault settlement and horizontal convergence in section ZK18 + 168 are shown in Fig. 17b, c. Similarly, the vault settlement and horizontal convergence in the Grade III and IV rock sections constructed with the HB method exhibit a rapid increase followed by a stable trend after excavating the upper bench of the tunnel. The final vault settlement for the Grade III and IV rock sections is 2.0 mm and 4.4 mm, respectively, and the final horizontal convergence for the Grade III and IV rock sections is 1.2 mm and 1.9 mm, respectively. The vault settlement and horizontal convergence in this study are well within the allowable deformation values, suggesting that the optimized excavation methods are suitable for the construction of the SLS tunnel.

## Conclusions

The present study focused on the Yangjiashan tunnel project in China to investigate the optimized excavation methods for shallow SLS tunnels in Grades III–V tuff stratum. Three sequential excavation methods were proposed and simulated using a finite difference program, including the "upper first and lower later" SD method, the CD method, and the HB method. The mechanical response characteristics of tunnel construction under these methods, such as rock deformation, rock pressure, and internal forces acting on the primary support, were comprehensively examined. Taking into account factors such as safety, cost, and schedule, the optimal construction method for SLS tunnels in Grades III–V rock was determined and validated through field tests. The following conclusions can be drawn:

1. Based on longitudinal settlement profiles, the longitudinal changes in vault settlement exhibit an S-shaped evolutionary pattern: a gradual increase in advanced deformation, followed by a rapid increase, and finally reaching a stable deformation stage. The construction method significantly influences the spatial deformation effect on the surrounding rock. The more excavation steps involved in the construction of the SLS tunnel, the smaller the disturbance extent and rock deformation during excavation.
2. The ratio of the vault settlement above the tunnel face for different excavation methods in Grades III–V rock accounts for 30–50% of the final displacement. To limit advanced convergence and maintain tunnel stability, it is necessary to implement advance reinforcement measures, such as advance small pipe grouting reinforcement, deep hole grouting reinforcement, and pipe shed support, before excavation when constructing SLS tunnels in soft or weak geological strata.
3. The influence of the construction method on the excavation response of SLS tunnels varies with rock conditions. The higher the quality of the surrounding rock, the smaller the influence of excavation methods on the deformation of the SLS tunnel.
4. The stress in the surrounding rock depends on the interaction between the rock and support and is closely related to the rock properties. When the surrounding rock is of poor quality, fewer excavation steps lead to more stress release, resulting in stress reduction and transfer to the deeper regions of the surrounding rock. Conversely, when the surrounding rock properties are good, fewer excavation steps and more stress release are more conducive to fully utilizing the self-bearing capacity of the surrounding rock.
5. The distribution of axial forces on the primary support in Grades III and IV rocks is similar to that in Grade V rocks, showing a distribution pattern of "large in arch waist and side walls, small in arch and inverted arch." The highest axial forces on the primary support in Grade V rock mass are approximately 2–3 times those in Grade III and IV rocks.
6. The results of the field monitoring show that the "upper first and lower later" SD method in Grade V rock mass and the HB method in Grade III to IV rock mass are reasonable. This study can provide technical references for the design and construction of SLS tunnels in similar conditions.

## Data Availability

The datasets used and/or analysed during the current study available from the corresponding author on reasonable request.
